# Diagnostic accuracy of adropin as a preliminary test to exclude acute pulmonary embolism: a prospective study

**DOI:** 10.1186/s12890-022-02156-y

**Published:** 2022-09-18

**Authors:** Serhat Orun, Aliye Celikkol, Batuhan Ilbey Basol, Elif Yeniay

**Affiliations:** 1 Emergency Medicine Department, Namık Kemal University Medicine Faculty, Tekirdağ, Turkey; 2 Medical Biochemistry Department, Namık Kemal University Medicine Faculty, Tekirdağ, Turkey

**Keywords:** Adropin, Biomarker, Emergency medicine, Acute pulmonary embolism

## Abstract

**Background:**

This study aims to investigate the diagnostic accuracy of adropin as a biomarker to exclude the diagnosis of acute pulmonary embolism (PE).

**Methods:**

Patients admitted to the emergency department of a tertiary health centre between August 2019 and August 2020 and diagnosed with PE were included in this prospective cohort study. The amount of serum adropin was determined in patients with (PE) and compared with that of healthy volunteers. Receiver operating characteristic analysis was performed with the obtained data, and the area under the curve (AUC) with a 95% confidence interval was determined. The parameters of diagnostic accuracy for PE were determined.

**Results:**

A total of 57 participants were included in the study (28 controls and 29 PE patients). The mean adropin level in the PE group was 187.33 ± 62.40 pg/ml, which was significantly lower than that in the control group (524.06 ± 421.68 pg/ml) (*p* < 0.001). When the optimal adropin cut-off value was 213.78 pg/ml, the likelihood ratio of the adropin test was 3.4, and the sensitivity of the adropin test at this value was 82% with specificity of 75% (95% CI; AUC: 0.821).

**Conclusion:**

Our results suggest that adropin may be considered for further study as a candidate marker for the exclusion of the diagnosis of PE. However, more research is required to verify and support the generalizability of our study results.

## Background

Acute pulmonary embolism (PE) occurs due to sudden vasoconstriction or obstruction of the pulmonary arteries, which most often results from a thrombus that originates from the deep leg veins [1, 2]. The disorder presents with frequent symptoms of sudden dyspnoea, stinging chest pain, tachycardia, cyanosis, syncope, and occasional lower extremity swelling accompanied by pain [2]. However, the condition is difficult to diagnose owing to a lack of specific symptoms and clinical signs. Although PE is an acute life-threatening condition, it is potentially reversible. Therefore, an early and accurate diagnosis of PE along with effective thrombolytic treatment is of the utmost importance.

Computed tomography pulmonary angiography (CTPA) is the gold standard method for the diagnosis of PE due to its easy accessibility, applicability and diagnostic accuracy [3]. However, the clinical application for this method has disadvantages due to the associated radiation exposure, the use of radio-opaque materials, and the need for a skilled operator. These disadvantages of radiographic imaging highlight the importance of using biomarkers and laboratory facilities for diagnostic purposes. Recent advances in biotechnology have engendered the detection of new biomarkers in the laboratory, such as adropin.

Adropin is a polypeptide consisting of a total of 76 amino acids, including the first 33 amino acids of the signal peptide, and has a molecular weight of 4999 Da [4, 5]. It is a small polypeptide and plays an important role in inflammation and some metabolic events [6]. Few studies have suggested the use of adropin as a biomarker in patients suffering from conditions associated with cellular hypoxia and destruction, such as acute myocardial infarction [7, 8].

This study aimed to determine the diagnostic accuracy of adropin to exclude the diagnosis of acute PE, which is characterized by cellular ischaemia and necrosis due to obstruction from a thrombus.

## Methods

### Ethics approval and informed consent

Ethical approval for this study was obtained from the noninvasive clinical research ethics committee of a local university (registration number—2019.185.10.06). Written informed consent was obtained from all patients and healthy volunteers who agreed to participate. The reporting of this study conforms to the STARD guidelines.

### Study design and participants

This prospective study was conducted in a university tertiary care hospital. Consecutive patients above the age of 18 years presenting to the emergency department (ED) between August 1, 2019 and August 1, 2020 who were diagnosed with pulmonary embolism based on the detection of a thrombus in CTPA were included in this study. Patients who were pregnant, had a history of malignancy, did not have CTPA imaging, referred to our centre with a confirmed diagnosis, and those who required cardiopulmonary resuscitation were excluded from the study. Healthy volunteers of similar age and gender were selected for the comparison group.

When the diagnosis of PE was confirmed in the ED, 5-ml blood samples were obtained from all included patients before the patients were admitted to the hospital and placed in red capped tubes (empty dry tubes, silicone-coated tubes). The tubes were then centrifuged at 3000 rpm for 15 min to separate the serum. The serum samples obtained were kept at -80 °C. Collected samples were brought to room temperature on the day of analysis, and serum adropin levels were analysed using the enzyme-linked immune sorbent assay method. Commercially available kits from Sino Gene Clon Biotech Co., Ltd. Hangzhou, China (catalogue no: SG-11594) were used for the study; the kits had intra-assay and interassay CV values of < 8% and < 10%, respectively.

CTPA imaging was used as the reference standard in this study. For imaging, patients were placed in the supine position. During imaging, patients were asked to take a deep breath and hold for 5–10 s. A GE Bright Speed Model 16 detector device was used for the procedure, and 0.8 cc/kg contrast agent was applied under 4 psi pressure.

Additionally, age, sex, presenting complaint, Geneva and Wells’ probability category score, bedside transthoracic echocardiography (TTE) findings, D-dimer, CRP levels, high-sensitivity troponin and arterial blood gas analysis for all patients were obtained and saved in their case report forms specifically designed for the study.

The laboratory technician evaluating the adropin test was not informed of the final diagnosis, clinical findings and CTPA results for each patient. The physician assigned to diagnose the patients in the ED and evaluate the CTPA images had not been informed about the adropin levels, which had yet to be evaluated.

To evaluate the efficacy of adropin, blood samples from diagnosed PE patients were compared to healthy volunteers with no active complaints and without any history of chronic disease. The treatment planned for any patient was not changed or delayed during the conduct of the study.

Before conducting the study, a similar study in the literature was used to estimate a sample size that would increase the power of the study above 80%. After the study began, it was confirmed that the sample size increased the power of the study above 80%.

### Statistical analyses

Statistical analyses were performed using Statistical Program for the Social Sciences version 18.0 (IBM, Inc.) and Analyse-it (Analyse-it Software, Ltd). The Kolmogorov‒Smirnov test was used to assess the normality of the distribution of the adropin levels and PE parameters, and the Mann‒Whitney U test was used to compare the PE and control groups. Pearson’s chi-squared test was used to determine the relationship between gender and PE variables and age and PE variables. Continuous variables were expressed as either the mean ± standard deviation or the median (min–max), and the Mann‒Whitney U test was used to determine relationships between continuous variables. Categorical values were expressed as absolute numbers and percentages. A value of *p* < 0.05 was considered statistically significant. Receiver operating characteristic (ROC) analysis was specified with 95% confidence intervals (95% CIs) and AUC values. The cut-off value was determined according to the likelihood ratio.

## Results

A total of 29 PE patients (average age = 64.48 ± 12.73 years) and 28 healthy controls (average age = 61.69 ± 27.22 years) were included in this study. In the PE group, 19 (65.5%) were females and 10 (34.5%) were males, while the control group had 12 females and 16 males. There was no statistically significant difference in age or sex between the PE and control groups (Table 1).

**Table 1:** Comparison of the demographic characteristics of the embolism and control groups

	n (%)	*p*
*Sex*		
PE group (Female)	19 (65.5%)	0.07
Control group (Female)	12 (42.8%)	
*Age*		
PE group	64.48 ± 12.73	0.12
Control group	61.69 ± 27.22	

PE, pulmonary embolism

The presenting complaints on admission for the 29 patients in the PE group were dyspnoea (n = 17, 58.6%), chest pain (n = 8, 27.8%), weakness (n = 2, 6.8%), tachycardia (n = 1, 3.4%), back pain (n = 1, 3.4%), and cough and haemoptysis (n = 1, 3.4%) (Table 2). In the PE group, the median value for D-dimer was 5.92 mg/L (0.81–35), the median CRP level was 39.5 mg/L (2.75–239), and the median troponin value was 55 ng/L (4–231). Furthermore, there was no correlation between adropin and D-dimer levels, CRP levels, or troponin levels (*p* = 0.2, *p* = 0.2, *p* = 0.2, respectively). The laboratory results for the PE patients are sown in Table 3.

**Table 2:** Complaints of PE group admission to the emergency room

Complaints	n (%)
Dyspnea	17 (58.6%)
Chest pain	8 (27.8%)
Weakness	2 (6.8%)
Tachycardia	1 (3.4%)
Backpain	2 (6.8%)
Syncope	1 (3.4%)
Cough	1 (3.4%)
Hemoptysis	1 (3.4%)
Fever	1 (3.4%)
Epileptic seizure	1 (3.4%)

PE, pulmonary embolism

**Table 3:** Laboratory findings of the PE group and additional adropin level of control group

Tests	Mean ± SD
D-dimer mg/L	9.64 ± 9.95
CRP mg/L	67.70 ± 64.38
Troponin ng/L	54.00 ± 45.39
Urea mg/dL	46.04 ± 29.01
Creatinine mg/dL	0.89 ± 0.25
WBC 10^3/ uL	11.03 ± 3.78
Hg g/dL	12.45 ± 2.17
Platelet 10^3/ uL	224.75 ± 87.03
Ph log[H +]-	7.45 ± 0.05
PO2mmHg	69.81 ± 23.83
PCO2mmHg	35.12 ± 8.70
Pt sn	13.97 ± 2.68
Aptt sn	23.92 ± 3.50
INR ınr	1.21 ± 0.24

PE, pulmonary embolism; WBC, white blood cell; INR, international normalized ratio

Among the 29 PE patients, 3 (10.3%) were in the low-probability group, 20 (69%) were in the intermediate-probability group, and 6 (20.7%) were in the high-probability group according to the Genova scoring system. The correlation between adropin levels and Genova score was statistically nonsignificant (*p* = 0.26). When the same patients were analysed using the Wells’ scoring system, 2 of them (6.9%) were at low risk, 25 (86.2%) were at intermediate risk, and 2 (6.9%) were at high risk. When the same patients were analysed using the European Society of Cardiology risk scoring system, 5 of them (17.2%) were at low risk, 14 (48.3%) were at intermediate risk, and 10 (34.5%) were at high risk. The correlation between scoring systems—adropin and D-dimer levels—was also nonsignificant (*p* > 0.05 in all groups) (Table 4).

**Table 4:** Adropin and d-dimer levels according to scoring systems groups in patients with PE

Tests	Genova categorization			
	Low	Intermediate	High	Between all categories
adropin	143.34 ± 35.04	189.27 ± 66.96	231.96 ± 97.53	*p* = 0.26
d-dimer	10.55 ± 0.43	9.47 ± 9.96	9.71 ± 12.54	*p* = 0.96

	Wells categorization			
	Low	Intermediate	High	Between all categories
adropin	177.09 ± 8.87	192.62 ± 77.16	218.79 ± 84.75	*p* = 0.56
d-dimer	3.0 ± 0	10.51 ± 10.48	5.16 ± 3.90	*p* = 0.73

	ESC categorization			
	Low	Intermediate	High	Between all categories
adropin	178.71 ± 71.10	175.98 ± 60.66	224.99 ± 84.32	*p* = 0.32
d-dimer	16.28 ± 5.32	11.64 ± 12.84	4.66 ± 3.11	*p* = 0.56

PE, pulmonary embolism; ESC, European Society of Cardiology

Among the TTE findings, the right ventricle/left ventricle diameter ratio (RV/LV) was below 0.9 in 9 (15.8%) of the PE cases. Right ventricle wall thickness (RVWT) was measured from the same points in all cases as standard; RVWT was less than 5 mm in 5 (17.2%) patients, 5 mm in 10 (34.5%) patients, and greater than 5 mm in 14 (48.3%) PE patients. Pulmonary artery systolic pressure (PASP) in 13 patients was below 15 mmHg, 15–30 mmHg in 6 patients, and ≥ 30 mmHg in 10 patients. The RV/LV ratio was below 0.9 in the entire control group. In addition, the RVWD value was5 mm in 4 participants and below 5 mm in 24 participants. Again, the PASP value was < 30 mmHg in the entire control group (Table 5).

**Table 5:** Comparison of adropin values and TTE findings between PE and control group

	Mean ± SD	*P* value
Adropin (PE group) pg/ml	187.33 ± 62.40	< 0.001
Adropin (control group) pg/ml	524.06 ± 421.68	
RV/LV ratio (PE group) cm	0.97 ± 0.17	< 0.001
RV/LV ratio (control group) cm	0.7 ± 0.08	
RVWT (PE group) mm	5.93 ± 1.79	< 0.001
RVWT (control group) mm	3.71 ± 0.71	
PASP (PE group) mmHg	26.68 ± 21.10	< 0.001
PASP (control group) mmHg	14.32 ± 2.22	

TTE, trans thoracic echocardiography; PE, pulmonary embolism; RV/LV, right ventricle/left ventricle; RVWT, Right ventricle wall thickness; PASP, pulmonary artery systolic pressure

The mean adropin level in the PE group was 187.33 ± 62.40 pg/ml, compared to 524.06 ± 421.68 pg/ml for the control group. The adropin level of the PE group was found to be significantly lower than that of the control group (*p* ≤ 0.001) (Table 5).

**Fig. 1:**
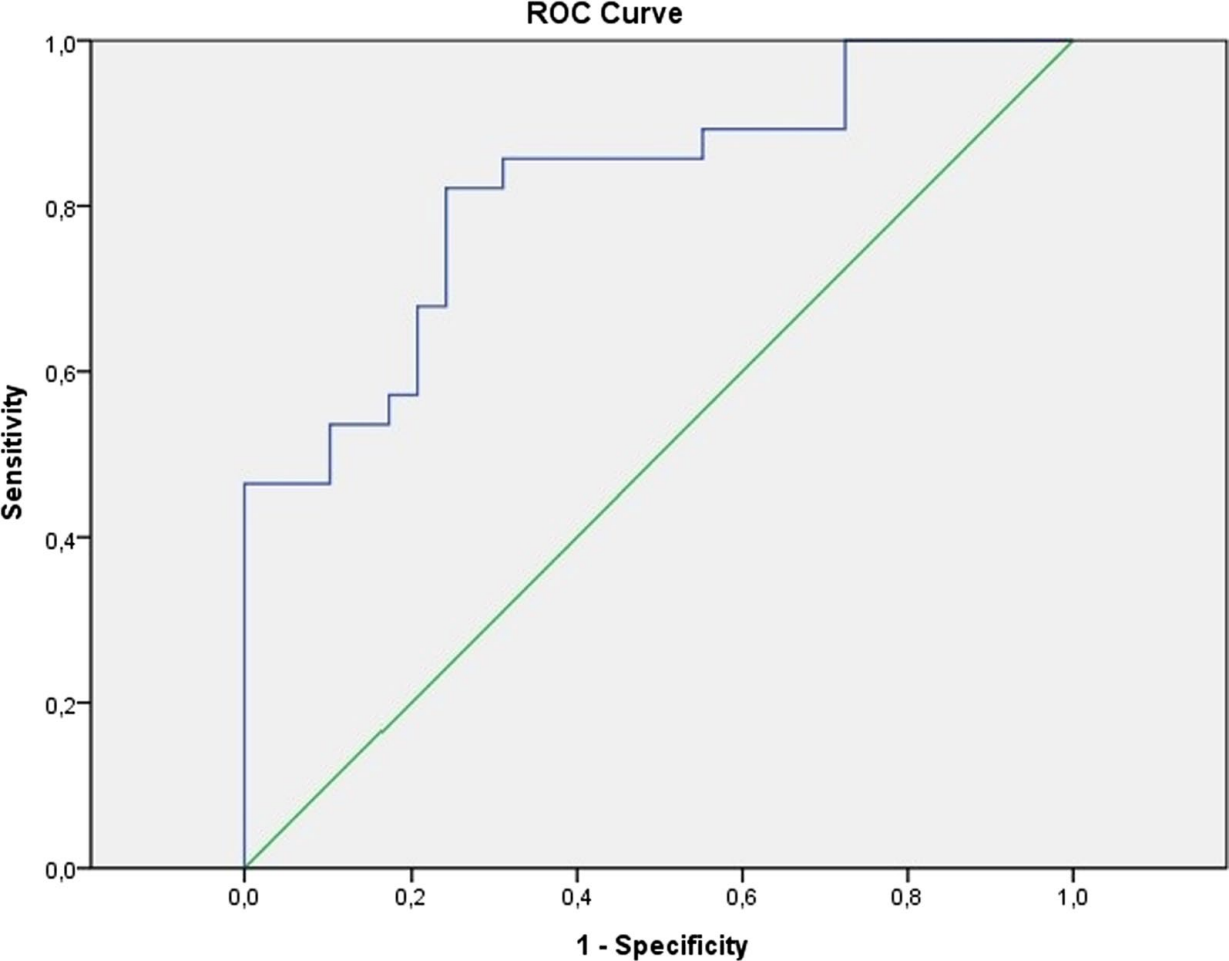
ROC curve of adropin value at excluding pulmonary embolism (blue curve). 95% CI, AUC: 0.821 (0.71–0.93)

ROC analysis was performed to determine the optimal adropin cut-off value, which was 213.78 pg/ml. At this adropin cut-off value, the sensitivity of the test was 82% (95% CI; AUC: 0.821), and the specificity was 75%. However, when the cut-off value was set at 304 pg/ml, the sensitivity of the test was 46%, and the specificity of the test was 96% (95% CI, AUC: 0.821) (Fig. 1).

After collecting the data, a sample size calculation was performed, and the power of the study was defined according to 29 patients and 28 controls. The effect size calculated on the data of the specified patients was determined to be 0.7985439. When the α error was accepted as 0.05, the power of the study was calculated as 84%.

## Discussion

This study investigates the performance of adropin as a biomarker for excluding the diagnosis of acute PE in patients presenting at the emergency department. Unlike D-dimer, serum adropin levels have been indicated to decrease in multiple scenarios and can be evaluated as potential biomarkers in conditions such as diabetes mellitus, arterial hypertension, obesity, sleep apnoea syndrome, and even osteoarthritis of the knees [9–12].

In recent guidelines, the increased awareness of various venous thromboembolic diseases, supplemented essentially with an increased availability of noninvasive imaging tests such as CTPA, encouraged clinicians to suspect PE more frequently and conduct a diagnostic study to confirm the diagnosis at an early stage [3]. However, the cost of unnecessary imaging and the desire to avoid the negative effects of radiation, while evaluating noninvasive diagnostic strategies for PE in recent times, suggest that PE should be safely excluded in the present patient population if they have a relatively low pretest possibility of having the disease [13]. In contrast, it is also emphasized that a positive test should have sufficient specificity to determine the indication for treatment, which is anticoagulant treatment in the case of PE [13].

In the ESC 2019 pulmonary embolism guideline, when PE is suspected at high risk in a haemodynamically unstable patient, bedside TTE or CTPA is recommended for diagnosis, depending on availability and clinical conditions [14]. The study that served as a reference for the guideline in making this prioritization was shared by Kucher et al. in 2003. In this study, it was stated that time-consuming imaging tests that increase the risk of sudden death and delay the initiation of reperfusion therapy can be avoided in patients with suspected pulmonary embolism with shock findings; TTE is a highly specific imaging modality that allows treatment decisions for pulmonary embolism in the presence of right ventricle dilatation and systolic dysfunction [15]. Kim et al. stated that TTE has critical importance in haemodynamic evaluation, and it may be useful in risk stratification, determination of therapeutic strategy, clinical decision making or evaluation of prognosis in PE [16]. In our study, bedside TTE was applied to all patients in the emergency department. We also think that TTE has critical importance in haemodynamically unstable high-risk patients with suspected PE and may help in time-critical decision making.

In the ESC 2019 pulmonary embolism guideline, it is recommended to use validated criteria for the diagnosis of PE in haemodynamically stable patients [14]. Stable patients were triaged again for CTPA imaging preference based on clinical risk and D-dimer level [14]. The guideline states that D-dimer measurement is recommended in ED patients with low or moderate clinical probability to reduce the need for unnecessary imaging and radiation and states that it rules out pulmonary embolism in 30% of outpatients [14, 17–19]. However, it is added that D-dimer should not be measured in the high clinical probability group, emphasizing that false negative results are reported in pulmonary embolism patients [14, 20]. In our study, D-dimer levels with standard cut-off values were measured, and clinical risk classification was performed in all suspected patients with stable haemodynamics. We also think that applying triage according to clinical probability and D-dimer results instead of direct CTPA in patients with suspected low- and intermediate-risk pulmonary embolism may reduce unnecessary imaging.

Awareness among clinicians of the importance of improving and simplifying acute pulmonary embolism has led to the testing of different biomarkers in studies on these issues. In the study by Talay et al., the diagnostic importance of mean platelet volume (MPV) on acute PE in the ED was investigated. In the results obtained from the study, it was stated that MPV (cut-off value 8.55 fl) has 82.2% sensitivity and 52.3% specificity in estimating patients with clinical suspicion of APE in the emergency department, with an AUC of 0.634 (95% CI 0.596–0.702) [21]. In the study of Insenser et al., it was determined that haptoglobin decreased in patients with severe pulmonary embolism. In addition, it is stated in the study that serum haptoglobin concentrations lower than 1 g/l show 80% sensitivity and 96% specificity in the diagnosis of high-risk PE. The AUC value stated in the study was 0.853 (95% CI 0.648–1.057) [22]. Apelin, fibulins, haemopexin, a2-macroglobulin, Ig a1-chain C region, TNF-α, HMGB1, neutrophil-to-lymphocyte ratio and albumin are among the other biomarkers whose effectiveness has been investigated in the diagnosis of pulmonary embolism [21–26]. miRNA studies on this subject have stated that these new methods and technologies can be deployed to discover new specific clinical diagnosis and risk stratification biomarkers, diagnostic schedules, and treatment protocols for pulmonary embolism [27, 28]. In our study, the relationship between adropin and PE was investigated (Fig. 1). Based on the results of our study, the optimum adropin cut-off value was determined to be 213.78 pg/ml. With this adropin cut-off value, the sensitivity of the test was 82% (95% CI; AUC: 0.821), and the specificity was 75%. If pulmonary embolism is to be excluded within a safer margin, a cut-off value of 304 pg/ml with 46% sensitivity and 96% specificity may be preferred. Because all results produced by studies without external validation may overestimate sensitivity and specificity because of overfitting as well as the retrospective design, all of these biomarker studies should be confirmed by new prospective cohort studies.

The D-dimer test has long been used to exclude the diagnosis of venous thromboembolism and pulmonary embolism. However, it also has some important limitations. Linkins et al. stated in their study that the wide variation in the types and study characteristics of D-dimer assays means that study results from one assay cannot be predicted to another. They also emphasize that there is still much work to be done in translating the results of D-dimer studies into clinical practice [29]. Li et al. investigated the effectiveness of D-dimer in differentiating PE and tuberculosis-related pleural effusion (TPE) cases in their study. While it was stated in the study that D-dimer levels were elevated in most of the patients with TPE, they suggested a new cut-off value for D-dimer in differentiating these two diagnoses [30]. Sandama et al. also stated in their study that given the low specificity of the D-dimer test for detecting venous thromboembolism, diagnosticians would probably prefer the development of a new test that would quickly give a positive result in every venous thromboembolism case without the risk of false-positive results [31].

Very few studies in the literature have examined the relationship between adropin and D-dimer. In their study, Yang M et al. investigated the presence of coronary artery lesions in Kavasaki patients, stated that D-dimer and adropin levels showed a positive correlation [32]. However, Aydın P et al. examined diabetes mellitus and COVID 19 patients in their study and found a significant negative correlation between adropin and C-reactive protein, D-dimer and ferritin levels [33]. In our study, however, there was no correlation between adropin and D-dimer levels. More studies are needed to clarify the correlation between adropin and D-dimer.

However, there are studies that provide some promising data on adropin. Kaluzna M et al. reported that adropin levels were not altered significantly during haemodialysis [34], and adropin was described as a potential candidate marker for cardiac dysfunction in patients undergoing haemodialysis. Additionally, Maciorkowska M et al. confirmed a negative correlation of adropin with the progression of kidney failure [9].

In our results, no acute or chronic kidney failure was experienced by patients in the PE group, which may have become a contraindication for using contrast substances in these patients. However, in the future, with increasing evidence when patients with limited kidney function are suspected to develop acute PE, serum adropin may be a useful alternative to D-dimer.

However, it is not known exactly how adropin levels are affected by various systemic and nonsystemic diseases. Therefore, higher adropin levels may be beneficial for excluding pathology rather than associating lower adropin levels with diagnosis.

### Limitations

First, there was a rather small sample size, so the results and conclusion should be viewed with caution. The cut-off value obtained from our study could not be validated in an external population, which was one of the major limitations of our study. Therefore, the cut-off value obtained from our study should be confirmed by new prospective cohort studies to confirm the sensitivity and specificity we calculated. The extent and type of inflammatory involvement in our PE patients and the influence of inflammation on adropin were not known. Additionally, despite excluding patients with a history of malignancy, the exclusion of patients with other comorbidities and the effect of such comorbidities on adropin levels limit the scope of generalizing our results. The sample size could not be increased for the control group as it is difficult to find healthy participants in advanced age.

## Conclusion

To conclude, our results suggest that adropin may be considered for further study as a candidate marker for the exclusion of the diagnosis of PE. However, since no studies are available that have investigated the diagnostic performance of adropin as a biomarker for PE, the results obtained from our study should not yet be considered generalizable.

## Data Availability

The datasets used and analyzed for this study are available from the corresponding author on reasonable request.

## Abbreviations

PE: Pulmonary embolism
ED: Emergency department
CI: Confidence interval
AUC: Area under the curve
CTPA: Computerized tomography pulmonary angiography
ROC: Receiver operating characteristic
TTE: Transthoracic echocardiography
MPV: Mean platelet volume
RV/LV: Right ventricle/left ventricle
RVWT: Right ventricle wall thickness
PASP: Mean pulmonary artery pressure
